# Renal and Salivary Gland Functions after Three Cycles of PSMA-617 Therapy Every Four Weeks in Patients with Metastatic Castration-Resistant Prostate Cancer

**DOI:** 10.3390/curroncol28050315

**Published:** 2021-09-23

**Authors:** Tim Wollenweber, Lucia Zisser, Elisabeth Kretschmer-Chott, Michael Weber, Bernhard Grubmüller, Gero Kramer, Shahrokh F. Shariat, Markus Mitterhauser, Stefan Schmitl, Chrysoula Vraka, Alexander R. Haug, Marcus Hacker, Markus Hartenbach, Sazan Rasul

**Affiliations:** 1 Department of Biomedical Imaging and Image-Guided Therapy, Division of Nuclear Medicine, Medical University of Vienna, 1090 Vienna, Austria; tim.wollenweber@meduniwien.ac.at (T.W.); lucia.zisser@meduniwien.ac.at (L.Z.); elisabeth.kretschmer-chott@meduniwien.ac.at (E.K.-C.); markus.mitterhauser@meduniwien.ac.at (M.M.); stefan.schmitl@akhwien.at (S.S.); chrysoula.vraka@meduniwien.ac.at (C.V.); alexander.haug@meduniwien.ac.at (A.R.H.); marcus.hacker@meduniwien.ac.at (M.H.); markus.hartenbach@me.com (M.H.); 2 Department of Biomedical Imaging and Image-Guided Therapy, Division of General Radiology, Medical University of Vienna, 1090 Vienna, Austria; michael.weber@meduniwien.ac.at; 3 Department of Urology, Medical University of Vienna, 1090 Vienna, Austria; bernhard.grubmueller@meduniwien.ac.at (B.G.); gero.kramer@meduniwien.ac.at (G.K.); shahrokh.shariat@meduniwien.ac.at (S.F.S.); 4 Department of Urology, Weill Cornell Medical College, New York, NY 10065, USA; 5 Department of Urology, Second Faculty of Medicine, Charles University, 15006 Prague, Czech Republic; 6 Institute for Urology and Reproductive Health, I.M. Sechenov First Moscow State Medical University, 119991 Moscow, Russia; 7 Department of Urology, University of Texas Southwestern Medical Center, Dallas, TX 75390, USA; 8 Ludwig Boltzmann Institute Applied Diagnostics, 1090 Vienna, Austria; 9 Christian Doppler Laboratory for Applied Metabolomics (CDL AM), Medical University of Vienna, 1090 Vienna, Austria

**Keywords:** PSMA, prostate cancer, mCRPC, renal scintigraphy, salivary scintigraphy

## Abstract

Background: [^177^Lu]Lu-PSMA-617 radioligand therapy (PSMA-RLT) could affect kidney and salivary gland functions in metastatic castration-resistant prostate cancer (mCRPC) patients. Methods: We retrospectively analyzed clinical, renal, and salivary scintigraphy data and salivary [^68^Ga]Ga-PSMA-11 ligand PET scan measures such as metabolic volume and SUVmax values of 27 mCRPC men (mean age 71 ± 7 years) before and 4 weeks after receiving three cycles of PSMA-RLT every 4 weeks. Twenty-two patients additionally obtained renal and salivary scintigraphy prior to each cycle. A one-way ANOVA, post-hoc Scheffé test and Cochran’s Q test were applied to assess organ toxicity. Results: In total, 54 PSMA PET scans, 98 kidney, and 98 salivary scintigraphy results were evaluated. There were no significant differences for the ejection fraction, peak time, and residual activity after 5 min for both parotid and submandibular glands prior to each cycle and 4 weeks after the last cycle. Similarly, no significant differences in serum creatinine and renal scintigraphy parameters were observed prior to each cycle and 4 weeks after the last treatment. Despite there being no changes in the metabolic volume of both submandibular glands, SUVmax values dropped significantly (*p* < 0.05). Conclusion: Results evidenced no alterations in renal function and only minimal impairment of salivary function of mCRPC patients who acquired an intense PSMA-RLT regimen every 4 weeks.

## 1. Introduction

Prostate cancer is one of the most commonly diagnosed cancers and the second leading cause of tumor-related death in men [1]. Prostate-specific membrane antigen (PSMA) is a class II transmembrane glycoprotein expressed in all types of prostate tissues. Nevertheless, overexpression of PSMA has been found in prostate tumors including its metastatic cells and in metastatic castration-resistant prostate cancer (mCRPC), making it an ideal target for prostate cancer diagnosis and therapy [2,3,4,5,6]. Therefore, various small molecule PSMA ligands have been developed, labeled with either gamma or positron emitters for positron emission tomography (PET) diagnosis or with beta or alpha particles for radionuclide therapy [7,8,9,10,11]. While the initial growth of prostate cancer is still androgen-dependent and can be effectively treated with luteinizing hormone-releasing hormone (LHRH) agonists and antagonists or anti-androgen receptors (ARs), almost all patients eventually advance to mCRPC where these therapies are no longer effective [12,13,14]. Consequently, [^177^Lu]Lu-PSMA-617 targeted radionuclide therapy (PSMA-RLT) has shown in numerous studies, including TheraP and Vision studies [15,16], promising results in terms of good tolerability, a favorable response rate, and the fewest adverse effects and organ toxicities in treated mCRPC patients [17,18,19,20].

However, PSMA is not prostate specific and several other organs such as the kidneys, salivary glands, lacrimal glands, or small intestine also express PSMA [21]. Therefore, proximal renal tubules and salivary glands, among others, are considered critical organs in patients receiving PSMA-RLT [22,23,24,25]. In this context, it has been shown that a highly standardized therapy regimen of 4–6 therapy cycles at 8–10-week intervals did not exceed the International Commission on Radiological Protection critical dose for critical organs such as kidneys and salivary glands, and no significant nephrotoxicity occurred in 10 patients treated with PSMA-RLT [26]. Indeed, the results of previous studies on the effects of PSMA-RLT on the kidney and salivary gland were largely based on the results of clinical centers offering this treatment to mCRPC patients with different inhomogeneous therapeutic regimens consisting of 1–8 cycles of 2–8 GBq activity per cycle and with an inter-cycle interval of 6–12 weeks [17,27,28]. Previously, we have shown that a more intensive, highly standardized PSMA-RLT protocol applied at our clinical institution with a shorter interval of only four weeks between the cycles has good tolerability and favorable response rates, progression-free survival, and survival rates for patients with mCRPC [29,30]. Therefore, the purpose of this study was to evaluate the renal and salivary glands (parotid and submandibular) toxicity under this unique intensive treatment regimen using clinical and scintigraphy parameters in mCRPC patients who all equally received three cycles of highly standardized PSMA-RLT every 4 weeks.

## 2. Methods

### 2.1. Study Population

**Figure 1:**
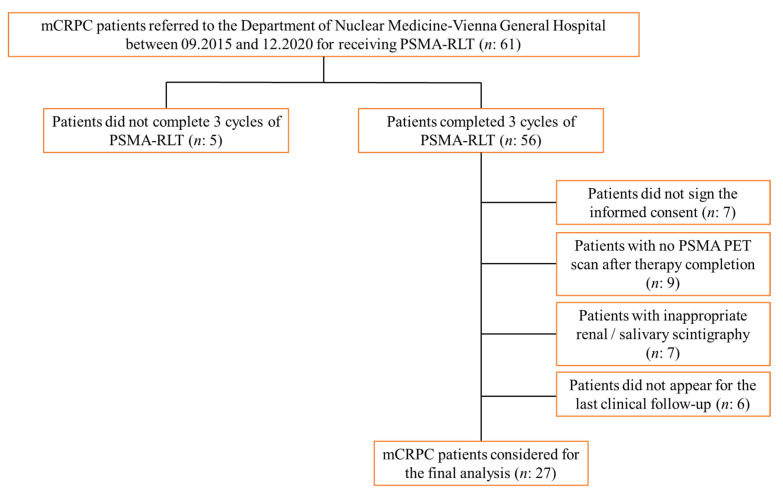
Flowchart for the selected mCRPC patients included in this study.

In this study, patients (*n*: 61) referred to the Department of Nuclear Medicine, Medical University of Vienna, Vienna General Hospital, between September 2015 and December 2020 to receive PSMA-RLT due to mCRPC were retrospectively evaluated. The therapy was performed in all patients with the recommendation of an interdisciplinary tumor board. The treatments were performed according to §8 of the Austrian Medicines Act (AMG). However, this analysis included only patients with properly and fully performed PSMA PET scans as well as salivary and renal scintigraphy (Figure 1). None of the patients studied underwent radiotherapy to the neck region. The studied patients had acquired a salivary and kidney scintigraphy directly before the first cycle and one month after the last (3rd) cycle of PSMA-RLT. Among them, a subgroup of patients additionally underwent salivary and kidney scintigraphy prior to each of the 3 therapy cycles. In addition, all patients underwent [^68^Ga]Ga-PSMA-11 ligand ([^68^Ga]Ga-PSMA) PET scans before the first cycle and 4 weeks after the last (3rd) therapy cycle. Clinical laboratory parameters including serum creatinine levels were measured in all patients before the start of each cycle and 4 weeks after the third cycle of therapy, and patients were requested to answer a questionnaire asking whether they suffer from dry mouth.

### 2.2. [^177^Lu]Lu-PSMA-617 Radioligand Therapy

The PSMA-617 precursor was obtained from ABX GmbH (Radeberg, Germany) and was labeled with [^177^Lu]Lutetium following procedures described previously [31]. In all patients, the therapy protocol consisted of 3 cycles of 7361 ± 293 MBq of PSMA-RLT administered intravenously every 4 weeks [30,32]. Prior to and after the slow intravenous administration of PSMA-RLT, each patient received 1000 mL of normal 0.9% saline infusion at 300 mL/h over 30 min. To protect the salivary glands, each patient received cold packs on the salivary glands 30 min before and up to 6 h after the therapy injection (p.i.), which were changed regularly.

### 2.3. Salivary Gland Scintigraphy

The salivary gland scintigraphy was performed on a double-headed gamma camera (Axis, Philips Medical Systems, Amsterdam, The Netherlands) equipped with a low energy all-purpose parallel hole collimator. The energy window around the 140 keV photopeak of [^99m^Tc]Technetium was 15%. Dynamic imaging was performed over 30 min after an intravenous administration of 102 ± 13 MBq 99mTc-pertechnetat in a 64 × 64-pixel matrix with 30 s per frame. Twenty minutes of p.i., p.i. = after the therapy injection, each patient received 5 mL lemon juice diluted with water (1:1). Patients were encouraged not to swallow the juice immediately but to hold it in the mouth for as long as possible and then swallow it without moving the head.

The data were analyzed using Hermes Hybrid 3D software (Hermes Medical Solutions, Stockholm, Sweden). For image analysis, a region of interest (ROI) was drawn over each salivary gland (left and right parotid, submandibular gland, oral cavity, and background). Time-activity curves were generated for each region. From these time-activity curves, the ejection fraction (EF) was defined as the percentage of the difference between the maximum count and the minimum count after stimulation divided by the maximum count [33,34,35]. Peak time was defined as the time after injection when the maximum count was reached. The residual activity (RA) at peak time plus 5 min was specified as the percentage of counts 5 min after the peak time divided by the maximum counts.

### 2.4. Kidney Scintigraphy

All kidney scintigraphy were also conducted on a double-headed gamma camera (Axis, Philips Medical Systems, Eindhoven, Nederland) equipped with a low energy all-purpose parallel hole collimator. The energy window around the 140 keV photopeak of [^99m^Tc]Technetium was 15%. After injection of 95 ± 11 MBq [^99m^Tc]-Mercaptoacetyltriglycine3 (MAG3), dynamic planar images from dorsal were acquired over 20 min (120 frames, 10 s per frame) in a 128 × 128 matrix. As for salivary gland scintigraphy, the data were analyzed using Hermes Hybrid 3D software (Hermes Medical Solutions, Stockholm, Sweden). For this, ROIs were drawn around each kidney. The background ROIs were then automatically drawn by the software.

From the generated time-activity curves, the relative function of the kidney was determined from the slopes of the right and left Patlak plots [36,37,38]. Since no blood samples were taken during the renal scintigraphy, the clearance parameters were determined using a camera-based method without blood or urine sampling. Therefore, we determined a measure of clearance similar to the methods that have been previously published [39,40]. Because the counts of the injected activity were not available, we could not express the clearance in terms of percent of uptake. Thus, we normalized the integral from 0.7 to 2 min over the renogram curves to the amount of the injected activity, which should be proportional to the injected counts using the same camera system for every patient.

### 2.5. PSMA-PET Imaging

Following our therapy protocol, all patients underwent [^68^Ga]Ga-PSMA PET examination prior to the initiation of the therapy and four weeks after the third treatment cycle. The scan was carried out 60 min after the application of 173.5 ± 16.3 MBq [^68^Ga]Ga-PSMA. Imaging was performed with four bed positions at 5 min scan time, thoroughly described in [41]. Accordingly, the parotid gland was not fully imaged in PET scans and, thus, a volume of interest (VOI) was generated only for the submandibular gland. To estimate the metabolic volume and the maximum standardized uptake value (SUVmax) of the submandibular glands, a cubic VOI was placed around the submandibular glands and then a threshold value of 10% of the maximum pixel value within the VOI was used for the delineation of the corresponding submandibular gland, as described previously in van Kalmhout et al. study [42].

### 2.6. Statistical Analysis

Descriptive statistical analysis was conducted with the software IBM SPSS Statistics version 24.0. The Kolmogorov–Smirnov test was applied to check the distribution of the values. Non-normally distributed data were presented as medians and ranges while normally distributed data were expressed as mean ± standard deviation. Other mentioned statistical analysis was performed using MedCalc v19.1 (Ostend, Belgium). One-way analysis of variance (one-way ANOVA) was used to test for statistically significant differences between the means of three or more groups. As a post-hoc test, the Scheffé test was conducted to find out which pairs of means were significant. Cochran’s Q test was used for evaluation of the results of the questioner concerning mouth dryness. A *p*-value lower than 0.05 was considered as statistically significant.

## 3. Results

### 3.1. Study Population

A total of 27 patients who underwent a proper [^68^Ga]Ga-PSMA PET scan as well as salivary and kidney scintigraphy prior to the first cycle and 4 weeks after the third cycle of PSMA-RLT were included in this study. The mean age of the patients was 71 ± 7 years. Prior to therapy, the median and range of creatinine level for all patients was 0.95 (0.71–1.16 mg/dL), respectively and of serum PSA level was 81.03 (5.91–3305 µg/L), respectively. The characteristics of the studied patients are summarized in Table 1. Of these patients, a subset of 24 patients underwent additional salivary and renal scintigraphy immediately before each PSMA-RLT cycle.

### 3.2. Salivary Gland Scintigraphy

**Figure 2:**
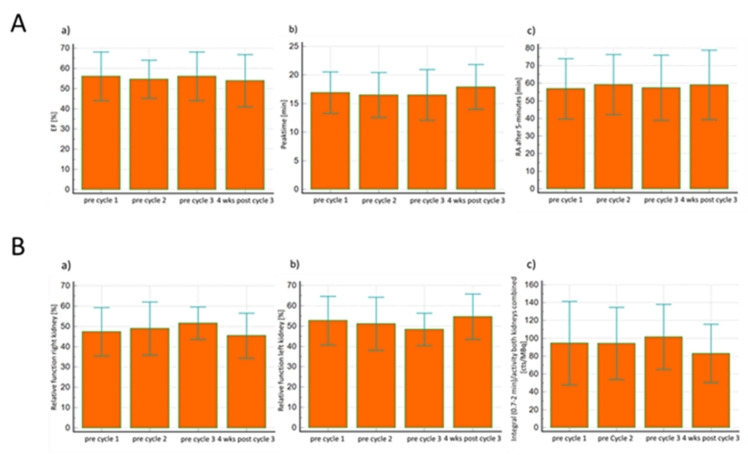

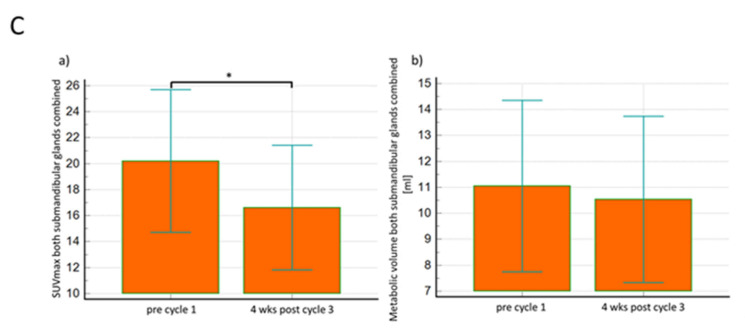
(**A**) Salivary gland scintigraphy prior to each three cycles of PSMA-RLT and four weeks after the third cycle. No significant difference in salivary gland function for percentage of ejection fraction (EF) (a) peak time (b) and RA after 5 min (c) for the whole parotid and submandibular glands prior to each three cycles of therapy and four weeks after the third cycle. wks.: weeks. (**B**) Kidney scintigraphy prior to each three cycles of PSMA-RLT and four weeks after the third cycle. Relative renal function such as slopes of the right (a) and left (b) Patlak as well as the integral of both kidneys combined from 0.7 to 2 min over the renogram curves normalized to the injected activity (c) did not reveal significant differences between the 3 therapy cycles and one month after the third cycle in all studied patients. wks.: weeks. (**C**) Quantification of the [^68^Ga]Ga-PSMA PET before the first cycle and 4 weeks after the last PSMA-RLT cycle. Significant reduction in values of SUVmax (a) without changes in metabolic volume (b) of the whole submandibular glands after receiving 3 cycles of PSMA-RLT. wks.: weeks.

In total, 98 salivary scintigraphy were performed. In all patients (*n*: 27), and as demonstrated in Table 2, there was no significant difference for the EF prior to as well as between each therapy cycle and four weeks after the 3rd PSMA-RLT cycle for the right and left parotid gland as well as for the right and left submandibular gland and for all glands together. Concerning the peak time in salivary scintigraphy prior to as well as between the three therapy cycles and one month after the last therapy cycle, there was no significant difference for the right and left parotid gland, or for the right and left submandibular gland. However, for all salivary glands combined, an ANOVA test yielded significant differences in the values of peak time before the start of therapy compared to the values four weeks after the last third treatment (*p* = 0.03), with the Scheffé test as a post-hoc test then revealing no significant differences in mean values between cycles, as shown in Table 2. In addition, there was no significant difference between the values of RA after 5 min for the left and right parotid glands as well as for the left and right submandibular glands, and for all glands together prior to the initiation of PSMA-RLT and four weeks after the last third cycle, all depicted in Table 2 and Figure 2a.

### 3.3. Kidney Function and Scintigraphy

There was no significant difference in mean creatinine levels between the 3 cycles of therapy and 4 weeks after the last cycle: (1st cycle 0.98 ± 0.28; 2nd cycle: 0.94 ± 0.27; 3rd cycle: 0.95 ± 0.28; four weeks after 3rd cycle: 1.02 ± 0.35; *p* = 0.58), as shown in Table 3. Furthermore, parameters of relative renal function acquired from renal scintigraphy such as the slopes of the right and left Patlak did not reveal significant differences between the first three therapy cycles and one month after the third cycle in all studied patients: (Patlak right: 1st cycle: 47.3 ± 11.9; 2nd cycle: 48.9 ±13.1; 3rd cycle: 51.6 ± 8.0; four weeks after 3rd cycle: 45.4 ± 11.1; *p* = 0.28) and (Patlak left: 1st cycle: 52.7 ± 11.9; 2nd cycle: 51.1 ± 13.1; 3rd cycle: 48.4 ± 8.0 four weeks after 3rd cycle: 54.6 ± 11.1; *p* = 0.28). There was also no significant difference for the integral of 0.7 to 2 min over the renogram curves normalized to the injected activity as a measure of clearance, as well as there being no significant difference between the values before the three cycles of therapy and the values four weeks after the third-to-last cycle: (both kidneys combined: 1st cycle: 94.5 ± 46.7; 2nd cycle: 94.3 ± 40.5; 3rd cycle: 101.5 ± 36.5; four weeks after 3rd cycle: 83.1 ± 32.7; *p* = 0.16), (right kidney: 1st cycle: 88.7 ± 42.3; 2nd cycle: 89.1 ± 38.8; 3rd cycle: 100.8 ± 31.7; four weeks after 3rd cycle: 77.0 ± 34.2; *p* = 0.20) and (left kidney: 1st cycle: 100.4 ± 50.5; 2nd cycle: 99.7 ± 43.0; 3rd cycle: 102.2 ± 41.8; four weeks after 3rd cycle: 89.2 ± 30.6; *p* = 0.67), see Table 3 and Figure 2b.

### 3.4. [^68^Ga]Ga-PSMA PET Imaging

Quantification of the [^68^Ga]Ga-PSMA PET images demonstrated significant difference in the SUVmax values for whole submandibular glands of both sides (20.2 ± 5.5 vs. 16.6 ± 4.8; *p* = 0.001) before the first cycle and 4 weeks after the third therapy cycle. Furthermore, SUVmax values of the left (20.5 ± 5.7 vs. 16.6 ± 4.8; *p* = 0.014) and the right (19.9 ± 5.4 vs. 16.6 ± 4.9; *p* = 0.03) submandibular glands significantly decreased four weeks after the third therapy cycle as compared to the SUVmax values before the therapy start, Table 4.

Concerning metabolic volume, on the other hand, results indicated no significant difference between the PSMA scan before and 4 weeks after the last therapy cycle, neither for the right nor for the left side, nor for the whole submandibular gland, see Table 4 and Figure 2c.

### 3.5. Questionnaire

Of the 27 patients, 21 completed a questionnaire concerning dry mouth prior to obtaining the first cycle, 20 prior to the second cycle, 14 prior to the third cycle, and 19 one month after the third cycle. Of these, four patients answered “yes” (19%) and seventeen (81%) answered “no” to the question about dry mouth before receiving PSMA-RLT. Before the second cycle, three men (15%) answered “yes” and seventeen (85%) answered “no”. Before the third cycle, two patients (14%) answered “yes” and twelve (86%) answered “no”. Four weeks after the third cycle, seven patients (37%) gave a “yes” response and twelve (63%) gave a “no” response. The results of the Cochran’s Q test indicated no significant differences on the question of dry mouth (yes or no) before obtaining each cycle and four weeks after the last third cycle, *p* = 0.19.

## 4. Discussion

Salivary glands feature a high expression of PSMA receptors and are, consequently, the organ with the highest absorbed radiation dose (1.0 ± 0.6 Gy/GBq) after PSMA-RLT [43]. This study is the first utilizing both salivary gland scintigraphy and [^68^Ga]Ga-PSMA PET images to assess the effect of an intensive PSMA-RLT regimen of three cycles of 7400 PSMA-RLT every four weeks on salivary gland function. Besides the fact that the values of salivary scintigraphy parameters such as EF, RA after 5 min, and peak time did not change significantly before and after treatment with this therapeutic regimen, there was only a small but statistically non-significant increase from 19% to 37% of patients reporting dry mouth. This is in good agreement with the mild and transient impairment of salivary glands previously noted in other studies under various PSMA-RLT regimens. Ahmadzarfar et al. reported dry lips in merely 20% of patients 2 weeks after receiving a cycle of 4.1–6.1 GBq [^177^Lu]Lu-PSMA-617 [31]. Other studies have either reported a temporary xerostomia or only a slight percentage of xerostomia (about 8.7%) in men who acquired 1–2 cycles of 4.1–7.1 GBq PSMA-RLT [44,45]. In addition, Scarpa et al. recorded xerostomia in three out of ten patients, which was transient in two patients and permanent in only one patient [46], and Kratochwil and colleagues described relevant xerostomia after three PSMA-RLT cycles in two out of thirty patients [27]. Although it was only mild (i.e., grade 1) or transient functional impairment, other earlier studies detected xerostomia in a large proportion of patients (87%) after up to four cycles of 7.5 GBq PSMA-RLT [43,47]. Hence, for the patients we studied, the mean peak time increased by approximately one minute when we compared values before and four weeks after the third cycle, indicating a slight impairment of salivary gland function after three cycles of therapy.

Furthermore, the results of the quantified [^68^Ga]Ga-PSMA PET scan displayed a significant decrease in SUVmax for the right and left submandibular glands and for both submandibular glands combined. This is in accordance with the findings of a previous study [46] in which a significant decrease in SUVmax was also evident for the submandibular glands after 2–3 cycles of 6.1 ± 0.3 GBq PSMA-RLT. Indeed, PSMA is known to be expressed on the epithelium of acinar gland cells and not on duct cells [48]. The decline in SUVmax can, thus, probably be explained by cell death resulting from salivary toxicity, which is accompanied by a loss of function. In contrast to Scarpa et al. who found a significant decrease in the volume of the submandibular glands from 7.5 mL to 6.2 mL, we did not find a meaningful change in the metabolic volume, which would have to be associated with an appreciable cell loss. This is also supported by the consideration that assuming an absorbed dose to the salivary glands of 0.8 to 2.5 Gy/GBq [49], the maximum cumulative dose under our therapy regimen of 3 cycles of 7.4 GBq only slightly exceeds the critical dose to the salivary glands of 26–50 Gy [43]. As mentioned in the introduction, PSMA-RLT can also be conducted with alpha emitters, in particular with [^225^Ac]Actinium. Here, salivary gland toxicity is also an important limiting factor of this therapy [50,51]. Thus, studies comparable to ours with salivary gland scintigraphy would be beneficial to assess the precise impact of such therapies on salivary gland function.

Regarding renal function, there was no significant change in mean creatinine levels during the entire duration of therapy and four weeks after the last cycle. This corresponds well with outcomes of several other studies that used other therapeutic regimens, in which no significant changes in renal function were also reported after PSMA-RLT. Among them is the recent work of Rosar et al. who demonstrated an increase in GFR determined by the MDRD formula after six cycles of PSMA-RLT with a median activity of 6.5 GBq [52]. Some studies described a dose-dependent mild renal function impairment of approximately 4.5% after 2–5 five cycles of PSMA-RLT with a mean cumulative [^177^Lu]Lutetium dose of 18.8 ± 6.7 GBq at 6–10 week intervals [53,54]. In other earlier studies, such as the study by Yadav et al., no nephrotoxicity was detected in 31 patients after 1.11–5.55 GBq [^177^Lu]Lu-PSMA-617 [55]. The proportion of patients with elevated cystatin C who had a higher diagnostic sensitivity than serum creatinine and could detect even moderate GFR limitation, increased from 25% at baseline to 58% after treatment in a study by Yordanova et al. [23], which might further indicate a slight reduction of only about 30% from baseline and the low burden of therapy on the renal function of mCRPC patients. Nevertheless, Rahbar et al. found no significant alteration in the median creatinine and median tubular extraction rate in male patients who experienced up to two doses of PSMA-RLT with a mean activity of 5.9 ± 0.5 GBq [56].

Essentially, even under our stricter therapy interval of only 4 weeks, there was no relevant nephrotoxicity, which was also confirmed by the results of renal scintigraphy. Specifically, the finding that the integral (0.7–2.0 min)/activity, as a surrogate parameter of renal function, did not change significantly suggests that no clinically relevant restriction of renal function occurs after PSMA-RLT. Likewise, the implication of [^51^Cr]Cr-EDTA GFR in the study of Hofman et al. to evaluate renal toxicity under the effect of PSMA-RLT revealed no renal toxic effects of this therapy on mCRPC patients after obtaining up to 4 cycles with a median activity of 7.5 GBq and a median time between treatment cycles of 6.1 weeks [47].

Despite the use of scintigraphy as a dependable investigation to assess salivary and renal function in a cohort of patients who all obtained a homogeneous therapy protocol with equal activity dose and interval between the cycles, the retrospective design of the study and the small sample size of patients analyzed might limit the findings of this research. Therefore, differences in the patient population concerning their pre PSMA-RLT treatments including chemotherapy, which might negatively affect renal function, and tumor stages could have influenced the incidence of treatment toxicity observed in patients included in this study. In addition, the short follow-up period of only 4 weeks after the last cycle of therapy is another issue that may hinder the conclusions of this present study.

## 5. Conclusions

Altogether, we concluded that the salivary gland and renal function of mCRPC patients were only slightly affected under the more restrictive treatment regimen of 7.4 GBq per cycle and an interval of only 4 weeks between cycles. This further supports the good tolerability and innocuity of PSMA-RLT in male patients with mCRPC, even though longitudinal studies of salivary gland function after PSMA-RLT might provide a better assessment of the long-term effects of this therapy.

## Figures and Tables

**Table 1:** Clinical characteristics of the entire studied mCRPC patients prior to receiving any PSMA-RLT.

Features	Values
Patients (*n*)	27
Age (mean ± SD) years	71 ± 7
Weight (mean ± SD) kilogram	85 ± 14
Karnofsky Score (*n*) %	
<80%	(9) 33
≥80%	(18) 66
ECOG-Index (*n*) %	
0	(0)
1	(25) 92.5
2	(2) 7.4
* PSA µg/L	81.03 (5.91–3305)
Hb (mean ± SD) g/dL	12.1 ± 1.6
Leucocyte g/L	6.9 ± 2.5
Thrombocyte (mean ± SD) g/L	242 ± 71
* Creatinine mg/dL	0.95 (0.71–1.16)
Previous treatments (*n*) %	
Enzalutamide/Abiraterone	(19) 70
Docetaxel/Cabazitaxel	(19) 70
Ra-223 (Xofigo^®^)	(11) 41
No chemo- or hormone or Ra-223 (Xofigo^®^)	(2) 7
Metastatic lesions (*n*) %	
cM1a	(5) 18.5
cM1b	(16) 59.3
cM1c	(6) 22.2

*n*: Number of studied patients; SD: Standard deviation; PSA: Prostate specific antigen; Hb: Hemoglobin; *: Data not normally distributed and presented in median and range.

**Table 2:** Function of salivary glands directly before each cycle and 4 weeks after receiving 3 cycles of PSMA-RLT.

Parameters Mean ± SD	1st Cycle (*n*: 27)	2nd Cycle (*n*: 22)	3rd Cycle (*n*: 22)	4 Weeks after 3rd Cycle (*n*: 27)	*p*-Value
Ejection fraction (%):					
All glands	56.0 ± 12.0	54.6 ± 9.4	56.0 ± 12.1	53.9 ± 12.9	*p* = 0.31
Right parotid	62.0 ± 11.6	59.3 ± 8.3	61.8 ± 10.8	57.1 ± 14.0	*p* = 0.28
Left parotid	58.7 ± 14.1	57.6 ± 10.4	58.5 ± 16.1	54.3 ± 15.9	*p* = 0.60
Right submandibular	51.1 ± 10.9	50.6 ± 9.3	52.4 ± 8.2	51.7 ± 10.6	*p* = 0.94
Left submandibular	52.3 ± 7.2	51.1 ± 6.6	51.4 ± 9.6	52.5 ± 10.3	*p* = 0.91
Peak time (minutes):					
All glands combined	16.9 ± 3.6	16.5 ± 3.9	16.5 ± 4.4	17.9 ± 3.9	*p* = 0.03 *
Right parotid	17.9 ± 2.5	17.8 ± 2.0	17.5 ± 3.8	18.6 ± 2.5	*p* = 0.50
Left parotid	18.2 ± 3.2	17.9 ± 2.8	18.1 ± 3.4	18.9 ± 3.9	*p* = 0.68
Right submandibular	15.7 ± 4.3	15.7 ± 5.1	14.6 ± 5.3	17.3 ± 4.0	*p* = 0.23
Left submandibular	15.9 ± 3.7	14.6 ± 4.3	15.9 ± 4.4	16.8 ± 4.7	*p* = 0.33
RA after 5 min:					
All glands combined	56.9 ± 17.2	59.2 ± 17.0	57.5 ± 18.5	59.0 ± 19.7	*p* = 0.64
Right parotid	47.7 ± 14.9	53.6 ± 17.9	50.2 ± 16.6	54.7 ± 21.0	*p* = 0.27
Left parotid	51.1 ± 17.2	55.1 ± 16.2	52.1 ± 18.0	54.9 ± 20.6	*p* = 0.73
Right submandibular	63.0 ± 13.5	63.3 ± 17.2	62.1 ± 18.8	64.1 ± 18.6	*p* = 0.98
Left submandibular	65.8 ± 16.1	65.0 ± 14.8	65.6 ± 17.0	62.3 ± 17.8	*p* = 0.84

SD: Standard deviation; RA: Residual activity; *: Scheffé-test demonstrated no significant differences between the different cycles.

**Table 3:** Serum kidney and isotope nephrogram ([^99^Tc]Tc-MAG_3_) parameters directly before each cycle and 4 weeks after receiving 3 cycles of PSMA-RLT.

Parameters Mean ± SD	1st Cycle (*n*: 27)	2nd Cycle (*n*: 22)	3rd Cycle (*n*: 22)	4 Weeks after 3rd Cycle (*n*: 27)	*p*-Value
Creatinine mg/dL	0.98 ± 0.28	0.94 ± 0.27	0.95 ± 0.28	1.02 ± 0.35	*p* = 0.58
* Relative function:					
Right	47.3 ± 11.9	48.9 ± 13.1	51.6 ± 8.0	45.4 ± 11.1	*p* = 0.28
Left	52.7 ± 11.9	51.1 ± 13.1	48.4 ± 8.0	54.6 ± 11.1	*p* = 0.28
# Integral:					
All	94.5 ± 46.7	94.3 ± 40.5	101.5 ± 36.5	83.1 ± 32.7	*p* = 0.16
Right	88.7 ± 42.3	89.1 ± 38.3	100.8 ± 31.7	77.0 ± 34.2	*p* = 0.20
Left	100.4 ± 50.5	99.7 ± 43.0	102.2 ± 41.8	89.2 ± 30.6	*p* = 0.67

SD: Standard deviation; *: Relative function = (Patlak/Slope Plot) %; **#**: Integral (0.7–2.0 min)/activity [counts/MBq].

**Table 4:** [^68^Ga]Ga-PSMA PET parameters of salivary glands directly before and 4 weeks after receiving 3 cycles of PSMA-RLT.

Parameters Mean ± SD	Prior 1st Cycle (*n*: 27)	4 Weeks after 3rd Therapy (*n*: 27)	*p*-Value
SUVmax.:			
All (both sides submandibular)	20.2 ± 5.5	16.6 ± 4.8	*p* = 0.001 *
Right submandibular	19.9 ± 5.4	16.6 ± 4.9	*p* = 0.03 *
Left submandibular	20.5 ± 5.7	16.6 ± 4.8	*p* = 0.014 *
Metabolic volume:			
All (both sides submandibular	11.1 ± 3.3	10.5 ± 3.2	*p* = 0.44
Right submandibular	11.3 ± 3.5	10.9 ± 3.3	*p* = 0.65
Left submandibular	10.8 ± 3.1	10.2 ± 3.2	*p* = 0.53

SD: Standard deviation; SUVmax: Maximum standard uptake value. *: Significant changes with Scheffé test before and 4 weeks after receiving 3 cycles of PSMA-RLT.

## Data Availability

The data presented in this study are available on request from the corresponding author.
